# Electrochemically Pretreated Carbon Microfiber Electrodes as Sensitive HPLC-EC Detectors

**DOI:** 10.1100/2012/295802

**Published:** 2012-04-30

**Authors:** Zdenka Bartosova, Daniel Riman, Petr Jakubec, Vladimir Halouzka, Jan Hrbac, David Jirovsky

**Affiliations:** ^1^Department of Analytical Chemistry, Faculty of Science, Palacky University Olomouc, 17. Listopadu 12, 771 46 Olomouc, Czech Republic; ^2^Department of Physical Chemistry, Faculty of Science, Palacky University Olomouc, 17. Listopadu 12, 771 46 Olomouc, Czech Republic

## Abstract

The paper focuses on the analysis and detection of electroactive compounds using high-performance liquid chromatography (HPLC) combined with electrochemical detection (EC). The fabrication and utilization of electrochemically treated carbon fiber microelectrodes (CFMs) as highly sensitive amperometric detectors in HPLC are described. The applied pretreatment procedure is beneficial for analytical characteristics of the sensor as demonstrated by analysis of the model set of phenolic acids. The combination of CFM with separation power of HPLC technique allows for improved detection limits due to unique electrochemical properties of carbon fibers. The CFM proved to be a promising tool for amperometric detection in liquid chromatography.

## 1. Introduction

Since its introduction in the 1970s, electrochemical detection in high-performance liquid chromatography (HPLC-EC) has developed into a reliable and versatile technique. HPLC-EC has found its way in many laboratories in various application fields because it represents an exceptionally powerful and progressive analytical tool for selective and ultrasensitive detection of various types of compounds. Among EC modes generally used, amperometry plays a major role in routine analysis.

A recent trend in HPLC is the miniaturization of the chromatographic system, which results in decreased consumption of both the sample and the mobile phase bringing significant economical and ecological benefits [1, 2]. In miniaturized analytical systems, not only concentration but especially the mass detection limits may improve significantly [3, 4]. Amperometric sensors can generally be easily adapted and can even profit when utilized in microscaled systems, unlike many other detection techniques (e.g., optical). For amperometry, a wide range of electrode materials is currently available, including noble metals, boron-doped diamond, and a broad spectrum of carbon-based working electrodes. Among carbon-based materials, cylindrical CFMs are widespread tools for monitoring neurotransmitters in vivo using amperometry and/or voltammetry [5]. They have also been applied to trace and ultratrace determination of both organic and inorganic electroactive species, namely, nitric oxide [6], drug monitoring in microenvironments [7], and so forth.

Surprisingly, few references can be found dealing with the use of individual cylindrical CFMs as detecting electrodes in HPLC-EC. As early as 1988, Hu and Kuwana [8] used cylindrical carbon fiber to detect traces of dansylated amino acids using HPLC-EC. Sagar et al. [9] studied determination of salbutamol in human plasma using HPLC-EC with CFM detector. White et al. [10] discussed the combination of rapid scan microvoltammetry performed on carbon microfiber electrode behind HPLC column. Few other papers appeared between 1994 and 2009 [11–16].

The cylindrical microelectrodes in general and cylindrical CFMs in particular benefit from the properties of microelectrodes (i.e., contribution of radial diffusion mass-transfer to Faradaic current resulting in improved faradaic-to-non-faradaic-current ratio) and, by providing relatively high currents, represent a powerful tool for amperometric measurements.

Additionally, microelectrodes exhibit lower sensitivity of the electrode response to irregularities in stirring (in the case of batch electrochemical experiment, e.g. amperometry) or irregularities in flow (when used in a flow system) compared to conventional millimeter-sized voltammetric electrodes [17].

Carbon fibers are materials made by pyrolysis of suitable precursors with polyacrylonitrile, pitch, and rayon being the most important. The common feature of the carbon fibers prepared in such a way is their composition from graphitic sheets which can be packed in the following three configurations: (i) radiating out from the center of the fiber (radial type), (ii) aligned in concentric arrangement (onion type), and (iii) distributed randomly (random type) throughout the fiber. The basal plane forms the backbone of the graphitic lattice, and the edge plane contains a significant population of oxygen-containing functional groups. Importantly, the final orientation of the graphitic structure largely determines the electrochemical performance. Because the edge plane of the graphitic sheet is more reactive than the basal plane, the application of a given carbon fiber electrode is dictated by its microstructure.

Besides its commercial unavailability, the infrequent use of CFM in HPLC-EC is mainly due to the fact that, observed analytical currents are extremely low with respect to CFM effective area, compared to currents commonly observed using conventional amperometric thin-layer cells. Thus, the use of a high-gain potentiostat is essential. The sensitivity of carbon fiber electrode can be substantially increased by suitable modifications of carbon fiber surface, which include electrochemical conditioning. The electrochemical conditioning (often referred to as “pretreatment”) is the preferred carbon fiber modification method due to its good reproducibility, high efficiency, and speed. The essence of the pretreatment is electrooxidation/electroreduction of the fiberis surface, yielding increased amount of surficial oxygen-containing functional groups (carbonyl, carboxyl, quinone, ether, ester, and hydroxyl), often denoted as carbon or graphitic oxide [18]. XPS and Raman studies [19, 20] have shown that carbonyl and hydroxyl groups are the predominate surface oxide functionalities on carbon. These moieties can modulate electron transfer rates for many electroactive species and can be specifically blocked using derivatization agents, for example, by Lucas reagent for surface hydroxyl groups and/or dinitrophenylhydrazine for surface carbonyls [21]. This way, the electrode surface properties can be adjusted for an optimum sensitivity towards the desired group of analytes. Beside specific modification of carbon fiber surface by formation of surficial oxygen-containing groups, nonspecific effects also occur, such as physical removal (etching) of the outer part of carbon fiber, resulting in fiber thinning. Accompanying the process is the increase in electrochemical surface area (typically about five-fold [22]), which is also beneficial for higher current densities achieved on “pretreated” fibers.

The performances of electrochemically pretreated carbon fibers were tested as detectors in narrow-bore HPLC-EC on a model mixture of phenolic acids. Phenolic acids are known to be effective natural antioxidants widely spread in plants and, thus, frequently determined in real samples of plant materials, food products, and so forth. As follows from their antioxidant nature, they can readily be electrochemically oxidized. HPLC-EC has already proven to be a very efficient technique in analysis of phenolic acids in natural products and other matrices [23–25].

## 2. Experimental

### 2.1. Materials

The standards of phenolic acids (gallic, protocatechuic, gentisic, 4-hydroxybenzoic, and caffeic) were purchased from Sigma (Sigma, St. Louis, MO, USA). Solutions of analytes (0.1 mg/mL) were prepared freshly by dissolution of phenolic acids in methanol, and work solutions were prepared by diluting the methanolic solution by the mobile phase. For the mobile phase preparation, disodium hydrogen phosphate, sodium perchlorate, and phosphoric acid (TraceSELECT purity) purchased from Fluka (Fluka AG, Buchs, Switzerland) were used. Methanol and acetonitrile (Merck, Darmstadt, Germany) were of gradient grade purity.

### 2.2. Microelectrode Fabrication

**Figure 1:**
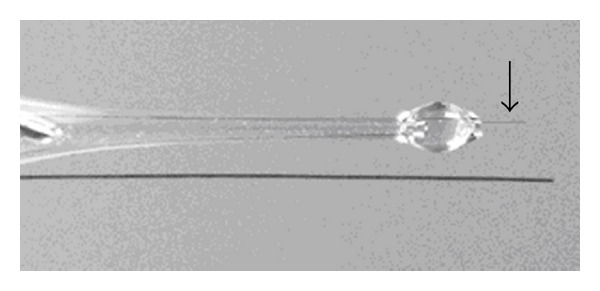
Carbon fiber microelectrode, comparison to human hair.

The procedure for microelectrode fabrication is as follows: carbon fiber is glued using conductive silver epoxy (Epotek H20E, Polytec, Germany) onto a copper wire and the junction is then cured at 150°C for 10 min. The fiber with copper contact attached is fitted into the glass capillary, about 10 mm of the fiber is left protruding from its contracted end. Both ends of the capillary are sealed using epoxy resin (CHS Epoxy 1200, Sindat Pilsen, Czech Republic). Prior to use, the protruding fiber is cut to the length of about 5 mm by lancet, and the fiber end of the electrode is briefly sonicated in dichloromethane in order to remove grease (Figure 1).

### 2.3. Electrochemical Measurements

Electrochemical measurements were performed using nanoampere electrochemical workstation (L-Chem, Czech Republic, http://www.lchem.cz/). Silver-silver chloride electrode (MF-2052, Bioanalytical Systems, USA) was used as a reference electrode. Platinum wire served as an auxiliary electrode.

The prepared microelectrodes were subjected to electrochemical pretreatment performed by cycling the electrode in 1% (w/w) NaCl solution between 0 and 2.9 V (versus Ag/AgCl) for 20 s, 50 Hz sine wave, followed by 5 s at constant potential–0.8 V and 5 s at 1.5 V.

Amperometry at a constant potential of 1000 mV versus Ag/AgCl in 20 mL of mobile phase was used to characterize the microelectrode sensors. In these experiments, the solution was stirred using a magnetic stirrer rotating at approximately 300 rpm. Background current obtained was allowed to decay until a stable baseline was achieved (usually approximately 1–5 min). The aliquots of methanolic stock solutions (1 mg/mL) of gallic and caffeic acids, respectively, were introduced into the cell using an autosampler (Titronic basic, Schott Instruments GmbH, Germany, adapted for computer control).

### 2.4. HPLC Measurements

The HPLC system consisted of an ESA isocratic pump (Model 582), (ESA Inc., Chelmsford, MA, USA) with a pulse damper, manual injector (Rheodyne, Cotati, CA, USA) equipped with a 2.5 *μ*L loop, and an ESA coulometric detector Coulochem III (used as a high-gain potentiostat in this study) with a guard cell (Model 5020) installed prior to the injector (all ESA Inc., Chelmsford, MA, USA). An L-Chem silver-silver chloride microelectrode was used as a reference electrode (L-Chem, Czech Republic, http://www.lchem.cz/). Chromatographic station clarity (DataApex, Prague, Czech Republic) was used for chromatogram recording.

The samples were introduced into the system by a glass 10 *μ*L syringe (Hamilton, Reno, NV, USA). All connecting fittings, ferules, and tubings were of PEEK. An Ascentis C18-A 3 *μ*m 100 × 2.1 mm i.d. (Supelco, Bellefonte, PA, USA) HPLC column was used.

The mobile phase consisted of 50 m*M* disodium hydrogen phosphate + 20 m*M* sodium perchlorate/acetonitrile (85/15, v/v), final mobile phase pH 3.0 was set up by concentrated phosphoric acid. The mobile phase was vacuum-filtered through a 0.2 *μ*m porous filter (Supelco, Bellefonte, PA, USA) and degassed by helium sparging prior to use. The flow rate was 0.2 mL/min.

The potentiostat set up gain ranged from 200 pA/V to 100 nA/V. The guard cell potential was set to +800 mV (versus Pd/H_2_) to eliminate traces of eventual electroactive contaminants from the mobile phase.

## 3. Results and Discussion

### 3.1. The Electrode Pretreatment and Its Impact on the Electrode Analytical Response

**Figure 2:**
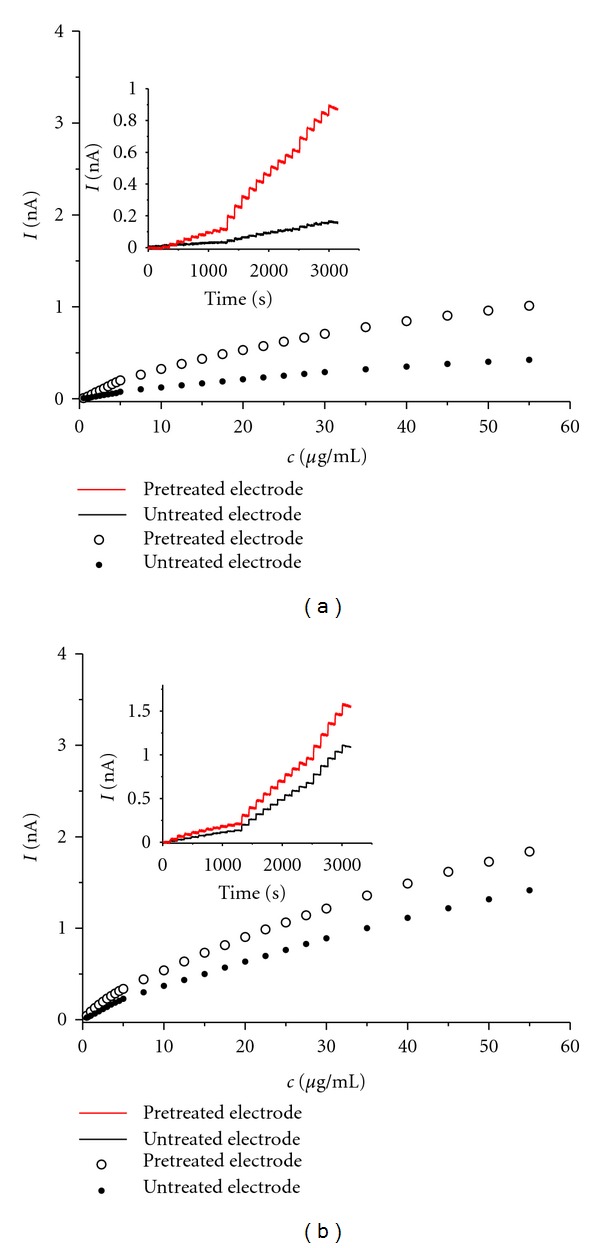
Calibration curves for gallic (a) and caffeic acid (b) on pretreated and native carbon fiber electrodes. Inset: actual amperometric response curves determined in stirred mobile phase. Applied potential: 1200 mV (versus Ag/AgCl).

The pretreatment protocol we adopted is widely used for both the construction of nitric oxide sensors [26–29] and the construction of dopamine/ascorbic acid sensitive sensors for in vivo measurements [22, 30–32]. In our previous work [33], we studied the effect of pretreatment on nitric oxide, nitrite, ascorbate, and dopamine. For the above analytes, the sensitivities as well as detection limits were improved by factors of 6, 35, 54, and 4.5, respectively. In this work, the increase in the electrode sensitivity caused by application of pretreatment procedure was examined using amperometry at 1200 mV versus Ag/AgCl for gallic and caffeic acids representing individual types of phenolic acids further studied by HPLC-EC. The amperograms and corresponding calibration curves are presented in Figure 2. The electrode sensitivities are improved by factor of 5.5 and 1.5 for gallic and caffeic acid, respectively.

### 3.2. Utilization of CFM in HPLC-EC

The analytical capabilities of the prepared CFM under flow-measurement conditions as well as its suitability for electrochemical detection in narrow-bore HPLC were examined. For the purpose of testing the CFM in HPLC detection, the CFM with fiber length of 5 mm was partially introduced (~4 mm) into the capillary outlet (red PEEK, 127 *μ*m ID) after HPLC column. Both capillary and microfiber electrodes (by means of its glass body) were mounted to a piece (~30 × 30 mm) of a printed circuit board and fixed firmly to avoid any accidental positional changes.

**Figure 3:**
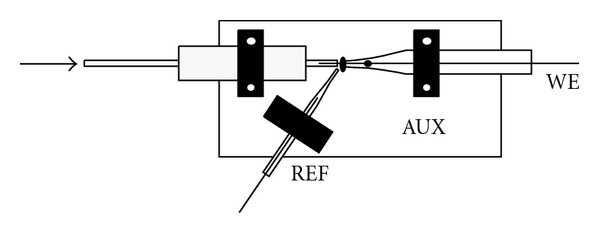
Scheme of the electrode arrangement used for HPLC measurements. Effluent flow direction is indicated by the arrow.

A three-electrode arrangement was used: auxiliary large-surface electrode was realized by the copper foil of a printed circuit board, and a Ag/AgCl reference minielectrode was placed in close proximity of the end of the HPLC capillary (Figure 3). 

Interconnection of the electrodes was mediated by the running conductive effluent. The electrochemical detection cell was controlled by the ESA Coulochem III potentiostat operating in one-channel mode, and connected to electrodes via a self-made wiring.

A standard mixture of common phenolic acids (gallic, protocatechuic, gentisic, 4-hydroxybenzoic, and caffeic acid) was used to test the suitability of the carbon microfiber electrode as an amperometric sensor in HPLC detection. 

**Figure 4:**
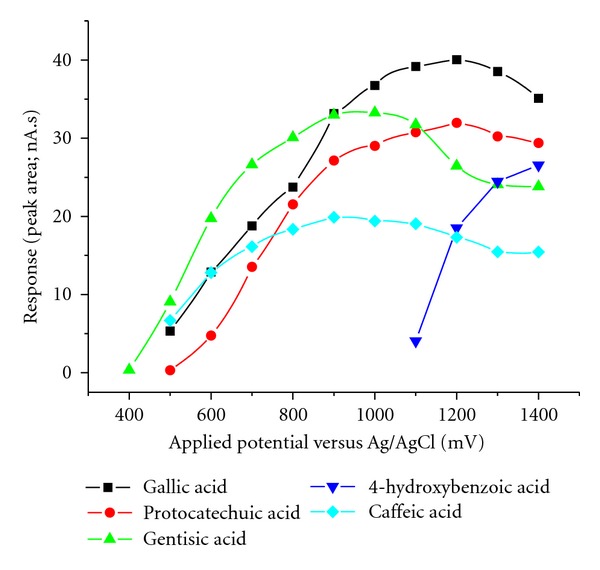
Hydrodynamic voltammograms of the phenolic acids used.

To assess optimum detection potential, hydrodynamic voltammograms of the studied analytes were obtained by consequent HPLC analyses of the model mixture (*c* = 500 ng/mL) at electrode potential settings between 0.3 and 1.4 V (Figure 4). As can be seen from the plot, individual phenolic acids differ in redox properties. A potential value of 1200 mV has been selected for the further HPLC experiment, as a starting point for simultaneous detection of analytes selected.

Even at high positive potentials applied to the working microfiber electrode, no adverse effects on either the signal background or the electrode performance were observed, proving their stability under these conditions. Presumably, even higher potential values can be applied to CME used. 

Calibration curves using HPLC conditions mentioned above were obtained for the analytes studied. Working potentials +1200 mV (versus Ag/AgCl) for gallic, protocatechuic, gentisic, and caffeic and +1400 mV (versus Ag/AgCl) for 4-hydroxybenzoic acid, respectively, were used. The principal calibration parameters as well as limits of detection (LODs) calculated are summarized in Table 1. LODs were obtained using equation LOD = 3.3*σ*/*b*, where *σ* was the standard deviation of the mean value for 5 signals using blank and *b* was the slope of the calibration curve.

**Figure 5:**
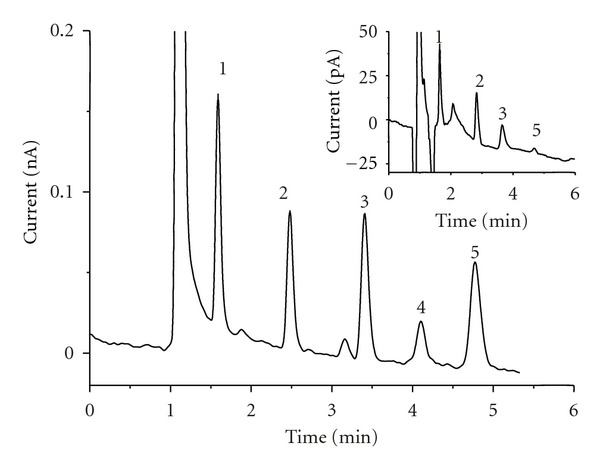
Chromatogram of standard mixture of phenolic acids. Conditions: amperometric detection at 1200 mV (versus Ag/AgCl), for other conditions see Section 2; analyte concentration: 10 ng/mL each. Inset chromatogram: analysis of 1 ng/mL concentration (2.5 pg on-column each acid). Legend: 1: gallic acid, 2: protocatechuic acid, 3: gentisic acid, 4: *p*-hydroxybenzoic acid, and 5: caffeic acid.

HPLC-EC analyses within picomolar concentration range are shown in Figure 5.

At relatively high working potentials (+1200 mV versus Ag/AgCl), the background noise remained at very low levels and, moreover, exhibiting a rather high-frequency character. After switching the working potentials, the electrode reacquisition proceeded fast, causing no significant delay in between consecutive measurements. Hydrodynamic characteristics of the used arrangement seemed to have no deteriorating effects on both the chromatographic resolution and the peak symmetry. As amperometric detection efficiency increases with enhanced mass transfer towards the electrode surface, utilizing the microfiber electrode in capillaries of smaller internal diameter than that used in this experiment can further improve detection limits and sensitivity. 

## 4. Conclusion

The experiments presented in this paper demonstrate the great potential of cylindrical CFMs when used as HPLC-EC detectors. Application of the developed electrode pretreatment protocol is, however, essential for obtaining the desired electrode analytical characteristics. Pretreatment not only results in improved sensitivity of the sensor but also allows for obtaining a stable baseline during long-lasting experiments. Artificially oxidized surface eliminates the problem of gradual oxidation of the sensor surface and allows HPLC-EC sensing at potentials higher than that used, for example, with glassy carbon-based thin layer HPLCEC cell, as shown in this work. 

The electrochemically treated CFM utilized in an HPLC system as described in this paper has proven to be a promising tool for sensitive amperometric detection in liquid chromatography. The designed amperometric detector exhibits an excellent sensitivity and signal stability, and its applicability for detection of other analyte classes will become a subject of further investigation. 

## Figures and Tables

**Table 1:** Main calibration parameters of the HPLC-EC.

	Intercept	Slope	*r* ^2^	LOD (pg/mL)
Gallic acid	0.50998	0.36435	0.99913	45.3
Protocatechuic acid	0.95112	0.41324	0.99912	39.9
Gentisic acid	0.28650	0.35023	0.99995	47.1
4-Hydroxybenzoic acid	−0.00785	0.02738	0.99946	1205.2
Caffeic acid	0.22750	0.34307	0.99998	57.7
